# Artemether-lumefantrine to treat malaria in pregnancy is associated with reduced placental haemozoin deposition compared to quinine in a randomized controlled trial

**DOI:** 10.1186/1475-2875-11-150

**Published:** 2012-05-03

**Authors:** Atis Muehlenbachs, Carolyn Nabasumba, Rose McGready, Eleanor Turyakira, Benon Tumwebaze, Mehul Dhorda, Dan Nyehangane, Aisha Nalusaji, Franois Nosten, Philippe J Guerin, Patrice Piola

**Affiliations:** 1 Department of Pathology, University of Washington, Box 357470, 1959 NE Pacific Street, Seattle, WA, USA; 2 Epicentre Mbarara Research Base, Mbarara, Uganda; 3 Shoklo Malaria Research Unit, Mae Sot, Tak, Thailand; 4 Faculty of Tropical Medicine, Mahidol University, Bangkok, Thailand; 5 Nuffield Department of Clinical Medicine, Centre for Tropical Medicine, University of Oxford, CCVTM, Oxford, UK; 6 Epicentre, Paris, France

**Keywords:** Malaria in pregnancy, Placental malaria, Artemisinin-based combination therapy, Quinine, Artemether-lumefantrine, *Falciparum*, Pathology, Histology, Randomized controlled trial, Haemozoin

## Abstract

**Background:**

Data on efficacy of artemisinin-based combination therapy (ACT) to treat *Plasmodium falciparum* during pregnancy in sub-Saharan Africa is scarce. A recent open label, randomized controlled trial in Mbarara, Uganda demonstrated that artemether-lumefantrine (AL) is not inferior to quinine to treat uncomplicated malaria in pregnancy. Haemozoin can persist in the placenta following clearance of parasites, however there is no data whether ACT can influence the amount of haemozoin or the dynamics of haemozoin clearance.

**Methods:**

Women attending antenatal clinics with weekly screening and positive blood smears by microscopy were eligible to participate in the trial and were followed to delivery. Placental haemozoin deposition and inflammation were assessed by histology. To determine whether AL was associated with increased haemozoin clearance, population haemozoin clearance curves were calculated based on the longitudinal data.

**Results:**

Of 152 women enrolled in each arm, there were 97 and 98 placental biopsies obtained in the AL and quinine arms, respectively. AL was associated with decreased rates of moderate to high grade haemozoin deposition (13.3% *versus* 25.8%), which remained significant after correcting for gravidity, time of infection, re-infection, and parasitaemia. The amount of haemozoin proportionately decreased with the duration of time between treatment and delivery and this decline was greater in the AL arm. Haemozoin was not detected in one third of biopsies and the prevalence of inflammation was low, reflecting the efficacy of antenatal care with early detection and prompt treatment of malaria.

**Conclusions:**

Placental haemozoin deposition was decreased in the AL arm demonstrating a relationship between pharmacological properties of drug to treat antenatal malaria and placental pathology at delivery. Histology may be considered an informative outcome for clinical trials to evaluate malaria control in pregnancy.

**Trial registration:**

REGISTRY: http://clinicaltrials.gov/ct2/show/NCT00495508

## Background

*Plasmodium falciparum* malaria in pregnancy is a major cause of morbidity and mortality for pregnant women and their offspring [1]. Artesunate monotherapy is more efficacious than quinine to treat severe malaria in Asian adults [2] and African children [3] and is now the recommended treatment [4]. The efficacy of artesunate and artemisinin-based combination therapy (ACT) in pregnancy has been well documented in Asia [5-8], however data on efficacy of ACT to treat malaria during pregnancy in sub-Saharan Africa is scarce [9-12]. The WHO currently recommends ACT for treatment of women in their second and third trimesters [4], yet quinine remains widely used even though the seven-day course is associated with more side effects and poor compliance [9,13,14]. Quinine may remain first line therapy due to greater availability, prescription habit and possibly lower cost (although this might be untrue thanks to increasing availability of subsidized ACT by various programmes, e.g. Global Fund, Presidents Malaria Initiative, and World Bank).

In an antenatal cohort of women in Mbarara, Uganda, a recent open label, randomized, non-inferiority trial of artemether-lumefantrine (AL) *versus* quinine demonstrated no difference in parasitological clearance rates corrected for re-infection by PCR genotyping [9]. Although there was no significant difference in clinical outcome, there was a trend towards decreased rates of low birth weight and pregnancy loss with AL [9].

The primary endpoints for treatment trials of malaria in pregnancy are PCR-adjusted cure rates (day 42) [15] and ultimately birth weight, however no data exists whether histopathology can be used to assess treatment efficacy. Histopathological endpoints are well established for clinical trials to treat chronic hepatitis B viral infection [16], to prevent renal allograft rejection [17], and may also function as surrogates for survival following chemotherapy [18]. During malaria, placental histopathology is strongly associated with birth outcomes [19-21], and has been utilized in a limited number of preventative trials: improved pathology was seen in two trials of intermittent presumptive therapy (IPT) compared to placebo [22,23], whereas no histological differences were observed with vitamin A administration [24] or in two trials examining differing IPT regimens [25,26].

**Figure 1:**
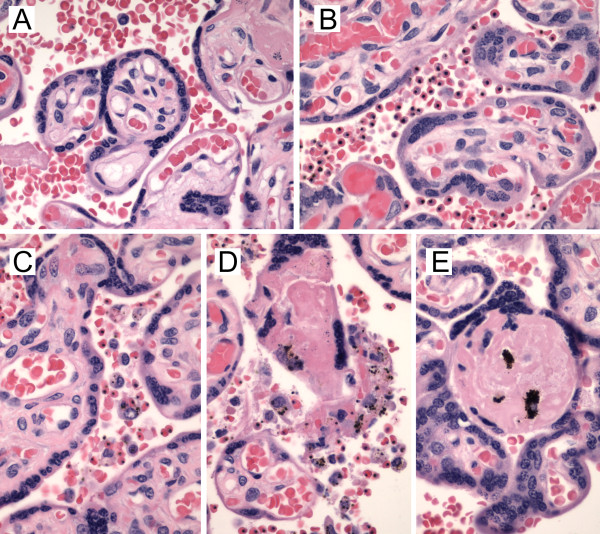
**Key features of placental malaria.** Haematoxylin and eosin stained placental sections from the Mbarara trial are shown at 600X. **A)** Normal placental histology. **B)** Parasitized erythrocytes sequester in the intervillous space. **C)** Monocyte-macrophages accumulate and phagocytose parasitized erythrocytes and haemozoin. **D)** Monocyte macrophages become enmeshed in fibrin and degenerate. **E)** Residual haemozoin persists in fibrin following successful treatment.

The histological hallmarks of placental malaria infection are parasitized erythrocytes, intervillous inflammatory infiltrate and haemozoin (malarial pigment) deposition in fibrin (Figure 1). In a treatment trial the majority of women are anticipated to be categorized traditionally as past or uninfected based on the presence or absence of haemozoin in fibrin [27]. A scoring system for placental malaria that includes semiquantitative analysis of haemozoin deposition in fibrin was developed to be appropriate for clinical trials with low incidence of parasitaemia at delivery [19], and here was applied to the current trial.

Artemisinin has a greater parasite reduction ratio than quinine [28], and unlike quinine is active on early ring stages. Both drugs are efficacious to treat uncomplicated malaria in pregnancy [9], however AL was hypothesized to lead to reduction of the cumulative parasite biomass within the intervillous space of the placenta and thus result in reduced downstream pathology. Haemozoin deposition and inflammation were assessed by histology for the randomized controlled trial from Mbarara to determine whether AL *versus* quinine to treat uncomplicated malaria in pregnancy was associated with reduced placental haemozoin and increased haemozoin clearance.

## Methods

Written informed consent was provided, and the study was approved by four regulatory boards: the Faculty Research Ethics Committee and the Institutional Review Board of the Mbarara University of Science and Technology, the Uganda National Committee for Science and Technology, and the Comits de Protection des Personnes (Ile de France XI, France). The use of specimens was approved by the University of Washington Human Subjects Division.

Women attended antenatal clinics from 2006 to 2009 at the Mbarara University of Science and Technology Hospital in Uganda, and were recruited to enter a cohort involving weekly screenings by blood smear. This is an area of meso-endemic transmission, with data for children under five demonstrating a 43% prevalence of *Plasmodium falciparum* determined by rapid diagnostic test (RDT) and corrected by blood smear in 2004 declining to 23% and 3% in rural and urban areas, respectively in 2010 [29]. Women with viable pregnancies >13weeks gestational age with positive blood smears by microscopy either asymptomatic or symptomatic but without complicated or severe malaria were eligible to participate in the open label, randomized, non-inferiority efficacy trial [9]. Women were directly observed to complete the seven-day course of oral quinine (10mg base per kg bodyweight every 8h for seven days) or the three-day course of oral AL (fixed-dose combination of 20mg and 120mg at 0h, 8h, 24h, 36h, 48h, and 60h, given with milk). Women with subsequent *P. falciparum* infection were treated depending on study arm such that they received the other study drug (quinine or AL). Women with non-*falciparum* infections received chloroquine. Women were followed in weekly antenatal clinics, with rapid diagnostic tests followed by blood smears. Subsequent parasitaemia was genotyped as described [9]. Intermittent presumptive treatment (IPT) was discontinued, and women were traced to their homes if they did not attend clinic.

**Figure 2:**
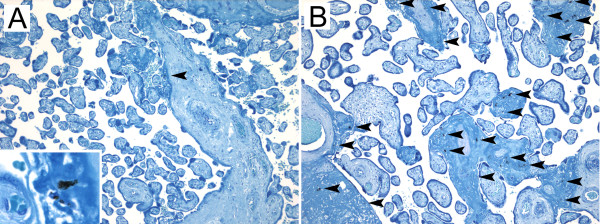
**Quantification of haemozoin deposition.** Giemsa stained sections are shown at 200X. **A)** A selected field from the placenta of a woman with infection at 162days prior to delivery demonstrating a focus of haemozoin (inset, 600X), which was quantified as 2% of HPF. **B)** A representative field from the placenta of a woman with infection at 77days prior to delivery demonstrating heavy haemozoin deposition quantified as 35% of HPF. Arrowheads: haemozoin deposits. HPF: 600X high power fields.

After delivery, placental biopsies were collected in neutral buffered formalin, stored for a period of one to four years, and were processed at University of Washington (UW) Medical Center Histology in 2010, with one haematoxylin and eosin and one Giemsa stained section per block. Formalin pigment was identified and these samples were excluded from analysis of pigment deposition or parasitaemia. Haemozoin deposition in fibrin and placental inflammation were scored on blinded sections as previously described [19]. Briefly, parasitized erythrocytes were identified by haemozoin and parasite cytoplasm within an erythrocyte in the absence of formalin pigment or nearby debris, intervillous inflammation was categorically graded, and haemozoin within fibrin was quantified as percentage of 600X high power fields positive for haemozoin (Figure 2). Fields were considered positive whether they contained single or multiple granules of haemozoin. A cut off value for haemozoin greater than 10% of high power fields (HPF) was previously associated with birth weight reduction and population distributions in cohorts from Tanzania and the Thai-Burma border [19].

Clinical data included treatment arm, gravidity, day of enrolment, level of parasitaemia, day of re-infection or recrudescence, and haemoglobin at delivery. The total number of antenatal visits prior to trial entry was not available. Analysis of birth weight and anaemia will be reported separately. Placental weights were not collected. Categorical variables were analysed by chi-square test or Fishers exact test. Continuous variables were analysed using the unpaired t-test, except for gravidity, parity and day of enrolment when the MannWhitney test was used. Parasitaemia at enrolment and the% HPF with haemozoin were log transformed prior to all analyses. Multivariate analysis was performed by ANOVA or logistic regression for continuous and categorical variables, respectively (Statview, SAS). Multivariate models incorporated variables expected to contribute to histological changes: duration between treatment and delivery, gravidity, re-infection or recrudescence, and parasitaemia at enrolment. Although there was a potential risk of confounding due to cross-over of study drug to treat subsequent parasitaemia, re-infection was included in the multivariate analysis because the infection at enrolment could have persisted from any time during pregnancy prior to enrolment, whereas re-infection would have occurred between weekly screenings and be promptly treated. Secondary analyses excluded women with re-infection from the multivariate models, and further included haemoglobin level at delivery, which has been associated with placental size [30]. Clearance curves were generated using Microsoft Excel (Microsoft).

## Results

**Figure 3:**
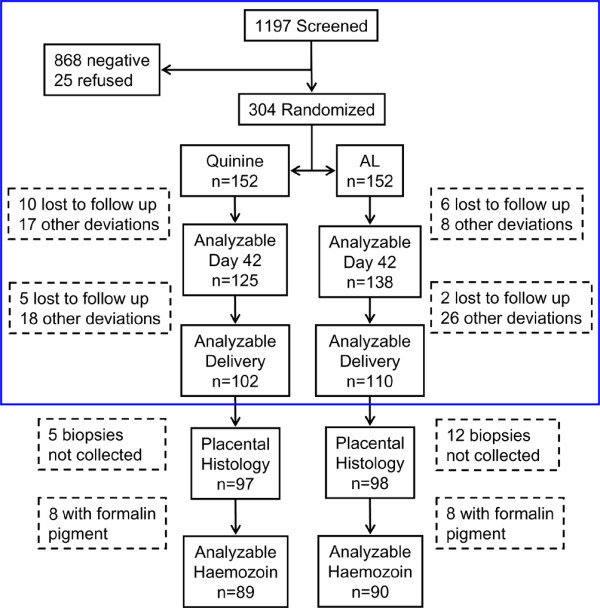
**Trial profile.** The blue box contains the original CONSORT trial flow chart, adapted from [9]. Placental histology was performed as a secondary analysis. AL- Artemether-lumefantrine.

Of 304 women in the trial, histology was available for 97 in the quinine arm, and 98 in the AL arm (Figure 3). There was no difference in maternal demographics or day of enrolment based on whether histology was available (Table 1). Of women with histology available, there was no difference by treatment arm for maternal demographics or infant outcome, although parasitaemia was slightly higher at enrolment in the quinine arm (Table 2).

**Table 1:** Demographic characteristics of women in the study separated by whether histology was available

		**With histology**	**Without histology**	**p**
Age (y)	Mean (SD); n	22.9 (5.0); 195	22.3 (4.7); 92	0.346
Parity	Median [range]; n	1 [0-7]; 195	0 [0-5]; 91	0.295
Gravidity	Median [range]; n	2 [1-8]; 195	2 [1-6]; 92	0.255
Birth weight (kg)	Mean (SD); n	3.04 (0.47); 169	3.00 (0.41); 70	0.552
EGA enrolment (weeks)	Mean (SD); n	23.8 (7.1); 194	22.5 (6.5); 92	0.144
Parasitaemia (per L)	Geometric mean [IQR]; n	1,546 [824-8,815]; 194	2,374 [451-6,453]; 92	0.092
EGA delivery (weeks)	Mean (SD); n	39.2 (3.4); 192	39.1 (3.9); 91	0.861
Days between treatment and delivery	Median [range]; n	104 [1-204]; 195	106 [1-201]; 92	0.604
Haemoglobin (g/dL)	Mean (SD); n	12.0 (2.2); 169	11.5 (2.3); 79	0.069

AL- artemether-lumefantrine; EGA- estimated gestational age; IQR- interquartile range.

**Table 2:** Demographic characteristics of women in the study separated by treatment arm for those with histology available

		**Quinine**	**AL**	**p**
Age (y)	Mean (SD); n	22.7 (4.6); 97	23.1 (5.3); 98	0.583
Parity	Median [range]; n	1 [0-6]; 97	1 [0-7]; 98	0.818
Gravidity	Median [range]; n	2 [1-7]; 97	2 [1-8]; 98	0.917
Birth weight (kg)	Mean (SD); n	3.02 (0.50); 85	3.06 (0.45); 84	0.525
EGA enrolment (weeks)	Mean (SD); n	23.7 (6.7); 96	24.0 (7.6); 98	0.800
Parasitaemia (per L)	Geometric mean [IQR]; n	2,006 [205-5,029]; 97	1,191 [504-10,335]; 97	0.075
EGA delivery (weeks)	Mean (SD); n	39.0 (3.8); 96	39.4 (2.9); 96	0.475
Days between treatment and delivery	Median [range]; n	105 [1-192]; 97	91 [1-204]; 98	0.994
Haemoglobin (g/dL)	Mean (SD); n	12.1 (2.4); 82	12.0 (1.9); 87	0.728

AL- artemether-lumefantrine; EGA- estimated gestational age; IQR interquartile range.

Among women with histology, re-infection rates with *P. falciparum* were similar between study arms: 12.2% (12/98) in the AL arm, with two women having three separate additional episodes each, and 13.4% (13/97) in the quinine arm with one woman having two separate additional episodes. By PCR genotyping [9], there was a single documented *P. falciparum* recrudescence in each arm. For non-*P. falciparum* infections (including *Plasmodium vivaxPlasmodium ovale, Plasmodium malariae*)*,* five women in the AL arm had single-species infections and one woman had mixed infection (with *P. falciparum*) and one woman had one of each. In the quinine arm, three women had single-species infections and one woman had a mixed infection (with *P. falciparum*). A single woman in the AL arm had taken sulphadoxine-pyrimethamine for IPT prior to enrolment. Of the five women in the clinical trial who did not complete the seven-day course of quinine prior to delivery, three women withdrew consent and two received rescue treatment but no specimens were available.

Considering the extent of prolonged storage, there were relatively few specimens with an obscuring amount of formalin pigment (16/195, 8.2%), which was associated with specimen dessication. Intervillous inflammation, but not malaria pigment or parasitaemia, could be reliably determined in specimens with obscuring formalin pigment. In the remaining specimens, 65.9% (118/179) had haemozoin, 8.2% (16/195) had intervillous inflammation, and 7.3% (13/179) had parasitaemia by histology; Table 2. Only a single case with high grade haemozoin deposition (>40% HPF) was present (quinine arm) and only a single case with massive intervillositis was present: she was within the AL arm and was enrolled two days prior to delivery of a stillborn infant. Of 13 women with parasitized erythrocytes by histology, six were under initial treatment at time of delivery (two in the AL and four in the quinine arm), three were placental blood smear positive (two in the AL arm [both re-infections] and one in the quinine arm [under initial treatment]), and the remaining five had parasites only detected by histology, with time of initial treatment ranging 70 to 100days prior to delivery (one of these women woman experienced three separate re-infections, with the most recent at 49days prior to delivery). There was one blood smear positive case (194 parasitized erythrocytes/L) that did not have parasitized erythrocytes detectable by histology; she had been treated with quinine 68days prior to delivery, and her delivery parasitaemia was confirmed as a re-infection by PCR genotyping.

Among all women with histology, a moderate or greater level of haemozoin deposition (>10% HPF) was independently associated with the proximity of the last malaria episode to delivery, lower gravidity, and re-infection (p<0.001, 0.022, and 0.014, respectively by logistic regression). Parasitaemia at enrolment significantly increased with proximity to delivery (R=0.285; p<0.001 by linear regression), and was increased in women with heavy haemozoin deposition by univariate analysis (p=0.001), however this association was non-significant in the multivariate analysis (p=0.095). Re-infection was not associated with differences in inflammation by histology, although the sample size was small. There were two samples from women who experienced PCR-confirmed recrudescence (one in each arm), however histology was compromised in each by formalin pigment. Among women who did and did not experience non-*P. falciparum* infections, no pathological differences were observed.

According to the treatment arm, there was no difference in the presence of formalin pigment, intervillous inflammation, parasitaemia, or absence of hemozoin by histology. However, the proportion of cases with moderate or greater levels of hemozoin (>10% HPF) were significantly reduced in the AL arm by univariate analysis (p=0.031) and by logistic regression after correcting for gravidity, day of enrolment, re-infection (yes/no), and parasitaemia at enrolment (p=0.028); Table 3. Results were similar when numbers of re-infections were included as a continuous variable. The effect of treatment arm on haemozoin level remained significant after the inclusion of haemoglobin at delivery (p=0.013), but was no longer significant after excluding the 22 women who experienced re-infection in the multivariate analysis (p=0.171).

**Table 3:** Histological analysis by treatment arm

		**Quinine**	**AL**	**p**	**p-adjusted**
Formalin pigment	n (%)	8/97 (8.2%)	8/98 (8.2%)	0.983	0.984
Haemozoin present	n (%)	60/89 (67.4%)	58/90 (64.4%)	0.675	0.954
Haemozoin (% of HPF)	Median [IQR]; n	3.3 [0-10.6]; 88	1.5 [0-5.7]; 90	0.090	0.101
Haemozoin >10% of HPF	n (%)	23/89 (25.8%)	12/90 (13.3%)	0.031	0.028
Inflammation	n (%)	8/97 (8.2%)	8/98 (8.2%)	0.999	0.974
Parasitized RBC	n (%)	5/89 (5.6%)	8/90 (8.9%)	0.566	0.241

Adjusted p-values are corrected for day of enrolment, gravidity, parasitemia at enrolment and reinfection. AL- artemether-lumefantrine; HPF- high power fields.

**Figure 4:**
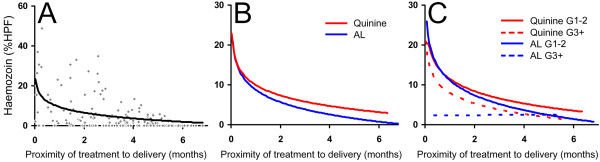
**Population haemozoin clearance curves.** Estimated curves derived from **A)** women in the trial (y=4.44ln(x)+9.71; R^2^=0.237), **B)** stratified by treatment arm, and **C)** treatment arm and gravidity. Excluding 22 women who experienced re-infection. HPF= high power fields.

Haemozoin level when quantified as a continuous variable (% HPF) was non-significantly decreased in the AL arm (p=0.090), and remained so after ANOVA (p=0.101). The longitudinal trial data allowed calculation of an estimated population haemozoin clearance rate for women with malaria treated prior to delivery, which was best fit to a logarithmic curve (Figure 4); women with re-infection were excluded. The amount of haemozoin increased with proximity of infection to delivery, however individual cases demonstrated much variation: for example one primigravid woman enrolled in the month prior to delivery had no detectable placental haemozoin deposition, whereas other primigravid women had high levels. Stratified by treatment arm, the magnitude of the curve was greater with quinine than AL although the slopes were similar. Because primigravid and secundigravid women had similar rates of low birth weight, placental parasitized erythrocytes and inflammation (data not shown) they were included in the same category for comparison to multigravid women (Figure 4C). No significant relationship with treatment arm was observed after excluding women with re-infection by ANOVA (p=0.310), however a trend was observed (p=0.082) when gravidity was included as a categorical variable, with initial clearance greatest in multigravid women treated with AL.

This trial involving women undergoing weekly malaria screenings since booking at antenatal clinics in Mbarara, Uganda, establishes the sensitivity of histology to detect haemozoin as a marker for past infection following successful treatment (Table 4), which decreased from 86% to 28% with proximity of infection ranging from one to six months prior to delivery. There was no difference by treatment arm. The maximum time between infection and delivery that haemozoin could be detected by placental histology in a woman without re-infection was 162days prior to delivery, at an estimated gestational age of 14weeks at enrolment (quinine arm, shown in Figure 2A).

**Table 4:** Sensitivity of histology to detect past infections

**Months prior to delivery**	**Haemozoin present; n (%)**
1	19/22 (86.4%)
2	23/28 (82.1%)
3	25/38 (65.8%)
4	23/34 (67.6%)
5	23/34 (67.6%)
6	5/13 (27.8%)
7	0/5 (0%)

Haemozoin presence stratified by month of last *Plasmodium falciparum* episode prior to delivery. Women who experienced re-infection are included in the month that re-infection occurred.

## Discussion

The overall results of the randomized controlled clinical trial demonstrated that AL was not inferior to quinine with a similar day 42 parasitological cure rate [9]. However AL was associated with a trend towards decreased rates of low birth weight and pregnancy loss. In the histological analysis, moderate and high-grade haemozoin deposition was decreased in the AL arm demonstrating a relationship between drugs used to treat an antenatal malaria episode and placental pathology at delivery.

**Figure 5:**
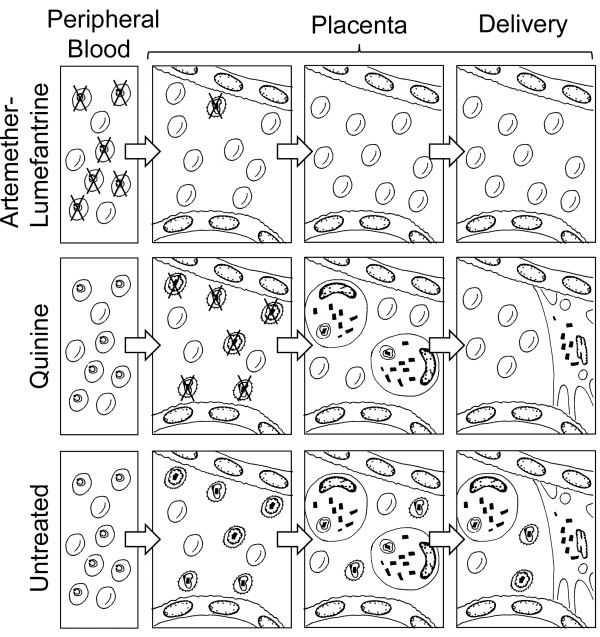
**Proposed model of increased ACT efficacy during pregnancy.** AL is active on early ring stages with a greater parasite reduction ratio, limiting parasite sequestration. Quinine is only active on mature parasites, which are sequestered in the placenta. Parasite sequestration leads to mononuclear cell infiltration and risk of poor pregnancy outcome. Haemozoin-laden macrophages become enmeshed in fibrin resulting in haemozoin deposition that persists until delivery. If women receive no or ineffective treatment, sequestered parasites, inflammation and haemozoin deposits persist until delivery.

Haemozoin deposits within fibrin are independently associated with birth weight during placental malaria [19], and they originate from monocyte-macrophages that phagocytose parasite material, subsequently become enmeshed in fibrin and degenerate. Monocyte-macrophages are a source of pro-inflammatory cytokines and associated with poor outcomes [31,32]. Based on these pathological data AL is hypothesized to be more clinically efficacious in pregnancy than quinine. Artemisinin derivatives are active at ring stages and have greater parasite reduction ratios compared to quinine [28] suggesting that ACT clinical efficacy could be linked to a greater reduction of sequestered mature-stage parasite biomass during treatment. In the placenta, this reduced sequestered parasite burden would result in less immune cell activation and associated phagocytosis which would be evident by reduced haemozoin in fibrin persisting until delivery (Figure 5). Haemozoin is biologically active and with a direct immunomodulatory effect *in vitro*[33], however it is unknown whether haemozoin embedded within placental fibrin exerts a biological effect during pregnancy or whether it is simply an inert marker of cumulative exposure to sequestered malaria parasites.

Haemozoin was not detected in approximately a third of cases, similar to previous reports of prompt and effective treatment of antenatal episodes resulting in no residual histopathology [34]. Further, comparatively low rates of intervillous inflammation and parasitaemia at delivery were observed in this trial, reflecting the efficacy of frequent antenatal screenings with prompt treatment of malaria in pregnancy. A much higher degree of pathology is seen in populations undergoing passive screening (consisting of IPT, bed net use and symptomatic treatment) although a formal comparison cannot be made across study sites due to differences in geography and study design. HIV status was not assessed in the trial, however considering that HIV is associated with increased rates of chronic PM [35] and delayed acquisition of protective immunity [36], HIV would be hypothesized to result in increased haemozoin deposition. The prevalence of HIV infection in antenatal clinics was previously reported to be 13% in the Mbarara region [37].

All women in the histological trial completed the directly observed seven-day course of quinine. Quinine is known to be associated with poor compliance [13] and data from this study would likely overestimate histological effectiveness of quinine in the population. In the clinical trial seven of eight interrupted treatments were in the quinine group [9]. Treatment failures in the AL arm most likely reflected altered pharmacokinetics during pregnancy of fixed dose lumefantrine [9] rather than resistance to AL.

Although there was possibility of confounding due to cross-over of study drug to treat re-infection, episodes of re-infection were deemed sufficiency different from the initial infection to include in the multivariate analyses. Re-infection occurred between weekly screening visits and was promptly treated whereas the infection at enrolment could have persisted for any length of time during pregnancy. In the absence of antenatal records available for review, these women were likely not screened prior to study enrolment. Exclusion of women with re-infection in secondary analyses demonstrated a non-significant effect by treatment arm, perhaps due to insufficient sample size.

The longitudinal trial design allowed for calculation of estimated haemozoin clearance rates. These curves hypothetically reflect the clearance of placental haemozoin following successful treatment over the course of gestation, analogous to parasite clearance curves from peripheral blood [38]. Curves were affected by treatment arm and parity, indicating a relationship between drug efficacy and immunity. Haemozoin clearance would be influenced by a combination of initial parasite burden, haemozoin dissipation through placental growth and perhaps biological clearance. Haemozoin levels increased with proximity of infection to delivery. Placental growth is most rapid in the third trimester where growth of the chorionic villi is likely to dissipate haemozoin deposits acquired earlier in gestation when the placenta was very small. Further, malaria in early gestation may limit placental growth [39], such that smaller placentas could be hypothesized to have higher levels of hemozoin. Placental weights were not collected in this trial and ideally future longitudinal studies would incorporate placental weight with ultrasound assessment of growth and measurements of placental haemozoin. Further, as a marker for malaria exposure, data on the sensitivity to detect past infections by histology (Table 4) would be useful for sample size calculations for programmatic studies to prevent or treat placental malaria prior to delivery.

As an alternative to histology, placental haemozoin content was previously analysed by spectrophotometry [40,41], and was similarly demonstrated to increase with proximity of infection to delivery. However spectrophotometry would also detect haemozoin within intact parasitized erythrocytes and macrophages, potentially confounding interpretation. For example, one subject in this study had a low level of placental haemozoin deposition (3.5% of HPF), consistent with effective treatment two months prior to delivery, however at delivery there was re-infection with 18% maternal erythrocytes parasitized with haemozoin-containing mature forms which would confound interpretation of treatment efficacy.

The specimens from this trial were processed in Seattle using state of the art histology equipment. Pathology is a crucial yet underfunded part of medical care in tropical countries [42] and although histology is labour intensive, prone to artefact and requires considerable expertise for interpretation, investment in laboratory and training of staff could strengthen local pathology services and generate long term benefit to the community. The placental sections generated in this study were of excellent quality and covered a wide range of pathology. This material was used to generate training slide sets distributed through the Malaria Research and Reference Reagent Repository [43] that will hopefully contribute to training and standardization in endemic areas. Improved local pathology systems would facilitate the assessment of placental effects of malaria in an era of changing transmission and increasing drug resistance.

In conclusion, in the randomized controlled trial AL was associated with lower rates of moderate to high-grade haemozoin deposition compared to quinine for treatment of uncomplicated malaria in pregnancy. Decreased haemozoin deposition in the AL arm likely reflects decreased cumulative sequestered parasite biomass. The results support the WHO guidelines for using ACT to treat malaria in the second and third trimester of pregnancy. Placental histology is a useful and sensitive tool to assess cumulative placental exposure to malaria to evaluate malaria control policy and implementation in pregnancy.

## Abbreviations

ACT: Artemisinin based combination therapy; AL: Artemether-lumefantrine; ANOVA: Analysis of variance; CONSORT: Consolidated standards of reporting trials; HPF: High power field; IPT: Intermittent presumptive treatment.

## Competing interests

The authors declare that they have no competing interests.

## Authors contributions

AM, RM, FN, PJG and PP designed the experiments. AM, CN, ET, BT, MD, DN, AN and PP performed the experiments. AM, RM and PP analysed the data. AM, RM and PP drafted the manuscript, and FN and PJG provided decisive comments. All authors read and approved the final manuscript.

## Author details

^1^Department of Pathology, University of Washington, Box 357470, 1959 NE Pacific Street, Seattle, WA, USA. ^2^Epicentre Mbarara Research Base, Mbarara, Uganda. ^3^Shoklo Malaria Research Unit, Mae Sot, Tak, Thailand. ^4^Faculty of Tropical Medicine, Mahidol University, Bangkok, Thailand. ^5^Nuffield Department of Clinical Medicine, Centre for Tropical Medicine, University of Oxford, CCVTM, Oxford, UK. ^6^Epicentre, Paris, France.
